# Impact of different dietary approaches on glycemic control and cardiovascular risk factors in patients with type 2 diabetes: a protocol for a systematic review and network meta-analysis

**DOI:** 10.1186/s13643-017-0455-1

**Published:** 2017-03-20

**Authors:** Lukas Schwingshackl, Anna Chaimani, Georg Hoffmann, Carolina Schwedhelm, Heiner Boeing

**Affiliations:** 1 German Institute of Human Nutrition Potsdam-Rehbruecke (DIfE), Arthur-Scheunert-Allee 114-116, 14558 Nuthetal, Germany; 2 Department of Hygiene and Epidemiology University of Ioannina School of Medicine, Medical School Campus, University of Ioannina, 45110 Ioannina, Greece; 3 Department of Nutritional Sciences, University of Vienna, Althanstraße 14, 1090 Vienna, Austria

**Keywords:** Diet, Type 2 diabetes mellitus, Network meta-analysis, Evidence synthesis

## Abstract

**Background:**

Dietary advice is one of the cornerstones in the management of type 2 diabetes mellitus. The American Diabetes Association recommended a hypocaloric diet for overweight or obese adults with type 2 diabetes in order to induce weight loss. However, there is limited evidence on the optimal approaches to control hyperglycemia in type 2 diabetes patients. The aim of the present study is to assess the comparative efficacy of different dietary approaches on glycemic control and blood lipids in patients with type 2 diabetes mellitus in a systematic review including a standard pairwise and network meta-analysis of randomized trials.

**Methods and design:**

We will conduct searches in Cochrane Central Register of Controlled Trials (CENTRAL) on the Cochrane Library, PubMed (from 1966), and Google Scholar. Citations, abstracts, and relevant papers will be screened for eligibility by two reviewers independently. Randomized controlled trials (with a control group or randomized trials with at least two intervention groups) will be included if they meet the following criteria: (1) include type 2 diabetes mellitus, (2) include patients aged ≥18 years, (3) include dietary intervention (different type of diets: e.g., Mediterranean dietary pattern, low-carbohydrate diet, low-fat diet, vegetarian diet, high protein diet); either hypo, iso-caloric, or ad libitum diets, (4) minimum intervention period of 12 weeks. For each outcome measure of interest, random effects pairwise and network meta-analyses will be performed in order to determine the pooled relative effect of each intervention relative to every other intervention in terms of the post-intervention values (or mean differences between the changes from baseline value scores). Subgroup analyses are planned for study length, sample size, age, and sex.

**Discussion:**

This systematic review will synthesize the available evidence on the comparative efficacy of different dietary approaches in the management of glycosylated hemoglobin (primary outcome), fasting glucose, and cardiovascular risk factors in type 2 diabetes mellitus patients. The results of the present network meta-analysis will influence evidence-based treatment decisions since it will be fundamental for based recommendations in the management of type 2 diabetes.

**Systematic review registration:**

PROSPERO 42016047464

**Electronic supplementary material:**

The online version of this article (doi:10.1186/s13643-017-0455-1) contains supplementary material, which is available to authorized users.

## Background

Type 2 diabetes mellitus (T2DM) is a chronic disease facing a growing number of cases worldwide, making it one of the most serious current health problems. Globally, in 2014 the prevalence of diabetes was estimated to be 9% among adults [1]. Raised blood glucose, insulin resistance, and low insulin sensitivity are the main characteristics of T2DM [2].

Dietary advice is one of the cornerstones in the management of T2DM [3]. The American Diabetes Association recommends a hypocaloric diet for overweight or obese adults with T2DM in order to induce weight loss [4]. However, there is limited evidence on the optimal approaches to control hyperglycaemia in T2DM patients [5]. The most recent nutrition guidelines of the American Diabetes Association indicate that there is uncertainty regarding the optimal proportion of energy coming from carbohydrates, protein, and fat for patients with T2DM [4]. Emerging evidence suggests that a diet high in monounsaturated fatty acids is associated with improved glycemic control [6–8]. Lifestyle changes can prevent the onset of T2DM as well as its progression. However, due to a wide array of diabetes-associated complications (such as cardiovascular diseases, retinopathy, and neuropathy), a variety of lifestyle changes should be explored to achieve a better protective effect [9, 10].

To our knowledge, up to date, no systematic review and network meta-analysis has been conducted to compare different dietary modifications in the management of T2DM. Some pairwise meta-analyses have been published comparing a higher vs. lower fat diet or different macronutrient compositions [11–13]. One of the most important questions that remains to be answered is which dietary approach offers the most benefits.

Therefore, our aim is to compare the efficacy of different dietary approaches on glycemic control and blood lipids in patients with T2DM in a systematic review including a pairwise and network meta-analysis of randomized trials.

## Methods and design

The review was registered in PROSPERO International Prospective Register of Systematic Reviews (https://www.crd.york.ac.uk/PROSPERO/display_record.asp?ID=CRD42016047464). The present systematic review protocol was planned, conducted, and reported in adherence to standards of quality for reporting systematic review and network meta-analysis protocols [14–17] (Additional file 1).

## Eligibility criteria

Studies will be included in the meta-analysis if they meet all of the following criteria:

### Types of studies

Randomized parallel or cross-over studies comparing different dietary approaches (e.g., dietary approach to stop hypertension; Mediterranean diet, vegetarian diet, Paleolithic diet, low-fat diet, low-carbohydrate diet, high protein diet, low-glycemic index/load diet) with a minimum intervention period of 3 months will be considered for this review.

### Types of participants

We will include studies with participants that are aged ≥18 years and are diagnosed with type 2 diabetes using the diagnosis criteria of the American Diabetes Association or other internationally recognized standards [18]. Studies including T2DM patients with hypertension and/or hyperlipidemia will be also included.

Studies including pregnant women, children, and adolescents, patients with normal glucose metabolism, and chronic renal disease will be excluded [19].

### Types of interventions

We will take into account all intervention trials that meet the abovementioned inclusion criteria and include at least one of the following intervention diets and a control, or other intervention diet.

Eligible types of diet will be the following:

- Low-carbohydrate diet (carbohydrates provide <30% total energy intake, high intake of animal and/or plant protein, often high intake of fat) [20];
- Low-fat diet (fat provide <30% of total energy intake, high intake of cereals and grains) [5, 20];
- Vegetarian diet (no meat, poultry, and fish);
- High protein diet (proteins provide >20% of total energy intake, high intake of animal and/or plant protein) [21];
- Mediterranean dietary pattern (fruit, vegetables, olive oil, legumes, cereals, fish, and moderate intake of red wine during meals) [22–26];
- Dietary approach to stop hypertension (high intake of fruits and vegetables, low-fat dairy, whole-grain cereals) [27];
- Low-glycemic index/load diet (glycaemic index and/or glycaemic load values must have been reported) [28, 29].

An intervention period of at least 3 months was chosen.

We will include either energy restricted diets, iso-caloric, or ad libitum diets.

The following dietary regimens will be excluded:

1. Intervention studies solely based on dietary supplements (e.g., vitamin C, vitamin E, calcium, potassium, garlic, soy protein) or single foods (e.g., nuts);
2. Intervention studies using dietary supplements as placebo (e.g., protein shakes);
3. Studies with an exercise/medication [30, 31] co-intervention that was not applied in all the intervention/control groups;
4. Interventions based on very low-energy diets (i.e., <600 kcal/day), since the Canadian Diabetes Association does not recommend the routine use of very low-calorie diets [32], while the American Diabetes Association suggests that these should not be used for more than 3 months without close supervision of a clinician [33].

**Fig. 1:**
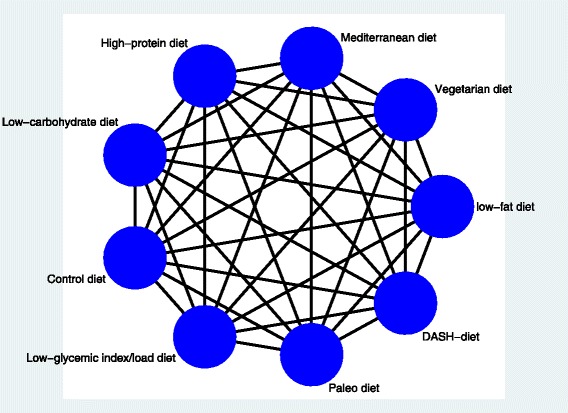
Network of all possible pairwise comparisons between the eligible dietary factors

Figure 1 shows the network of possible pairwise comparisons between the eligible dietary interventions.

## Outcome measures

The selected outcome measures represent the most utilized biomarkers for T2DM patients in clinical trials [34]. The primary outcome will be glycosylated hemoglobin (HbA1c); the following secondary outcome will be considered: fasting plasma glucose, body weight, total cholesterol, low-density lipoprotein cholesterol, high-density lipoprotein cholesterol, and triacylglycerol. All of these outcomes are evidence-based and validated risk factors for cardiovascular disease, the main cause of death for T2DM patients [35].

Blood samples should have been obtained after at least 8 h of overnight of fasting.

## Search strategy

The search will be performed by LS and CS, and differences will be resolved by discussion with a third reviewer (HB). We will conduct searches in PubMed, Cochrane CENTRAL, and Google Scholar. We will search for articles of original research using the following search terms for PubMed:

1. diet OR diet[MeSH Terms]
2. diabetes OR diabetes[MeSH Terms]
3. (low-carbohydrate[All Fields] OR high-carbohydrate[All Fields] OR low-fat[All Fields] OR high-fat[All Fields] OR low-protein[All Fields] OR high-protein[All Fields] OR vegetarian[All Fields] OR vegan[All Fields] OR Mediterranean[All Fields] OR DASH[All Fields] OR dietary approaches to stop hypertension[All Fields] OR glycaemic index[All Fields] OR glycaemic load[All Fields] OR Paleolithic[All Fields] OR low-calorie[All Fields] OR atkins[All Fields])
4. (glucose[MeSH Terms] OR glycemic[All Fields] OR glycaemia[All Fields] OR glycaemic[All Fields] OR glycemia[All Fields] OR HbA1c[All Fields] OR A1c [All Fields] OR glycated[All Fields] OR glycosylated[All Fields] OR lipids[MeSH Terms] OR body weight[MeSH Terms] OR cholesterol*[All Fields] OR lipid*[All Fields] OR lipoprotein*[All Fields] OR “high density”[All Fields] OR “low density” [All Fields] OR triacylglycerol*[All Fields] OR LDL[All Fields] OR HDL[All Fields])
5. (randomized controlled trial[All Fields] OR randomized[All Fields] OR clinical trials as topic[All Fields] OR placebo[All Fields] OR randomly[All Fields] OR trial[All Fields] NOT animals[All Fields])
6. (#1 AND #2 AND #3 AND #4 AND #5)

The search strategy will be adapted for each database.

Moreover, the reference lists from the retrieved original articles; systematic reviews and meta-analyses will be checked to search for further relevant studies. We will also conduct searches in Clinicaltrials.gov (http://clinicaltrials.gov/) and the World Health Organization International Clinical Trials Registry Platform to look for ongoing trials and opengrey (http://www.opengrey.eu/).

There will be no restrictions by language or publication year. Studies published in languages other than English will be translated by international scientists in our institute.

## Study selection process

Two reviewers will screen titles and abstracts of all the retrieved bibliographic records. Full texts of all potentially eligible records passing the title and abstract screening level will be retrieved and will be examined independently by two reviewers with the abovementioned eligibility criteria/exclusion criteria. Disagreements will be resolved by consensus or by adjudication of another author. A flow-diagram will outline the study selection process and reasons for exclusions for full-text articles. When a study was published in duplicate, we will include the version containing the most comprehensive information (e.g., longest follow-up duration and/or largest number of study participants).

## Data extraction

After determination of the study selection, two reviewers will extract the following characteristics: the first author’s last name, year of publication, study design (RCT: parallel or cross-over), diagnostic criteria for T2DM, mean baseline age (effect modifier), mean baseline BMI, mean baseline HbA1c, sex, description of the different dietary intervention arms, dietary assessment method, specification of the control group, type of diet (isocaloric vs. energy restricted vs. ad-libitum), drop outs, adverse events, and diabetes-associated complications during the study, and funding source using our own checklist. Piloting of the tool based on three studies will be performed. The following outcome measures will be extracted: post-intervention values or changes from baseline values with corresponding standard deviations for glycosylated hemoglobin, fasting plasma glucose, blood lipids: total cholesterol, low-density lipoprotein cholesterol, high-density lipoprotein cholesterol, triacylglycerol, and body weight (effect modifier).

## Risk of bias assessment

Full copies of the studies will be independently assessed by two authors for methodological quality using the risk of bias assessment tool from the Cochrane Collaboration [36]. The following sources of bias will be detected: selection bias (random sequence generation and allocation concealment), performance bias (blinding of participants and personnel), attrition bias (incomplete outcome data), reporting bias (selective reporting). Randomized controlled trials in nutrition research are often prone to inherent methodological constraints (e.g., they sometimes cannot be controlled with “true” placebos, but rather by a limitation of certain aspects of nutrient compositions, food groups, or dietary patterns).

Studies will be classified as being at low risk of bias if ≥3 out of a maximum of five risk of bias sub-items were rated with a low risk of bias by using the risk of bias assessment tool from the Cochrane Collaboration.

## Dealing with missing data

We will try to obtain relevant missing data from authors of the included RCTs (by e-mail). If the post-intervention values with the corresponding standard deviations are not available, the change scores with the corresponding standard deviations will be imputed, according the guidelines of the Cochrane Handbook [37].

## Evaluation of synthesis assumptions

### Data synthesis

#### Description of the available data

We will generate descriptive statistics for study and population characteristics describing the available data and some important variables (e.g., age, length of follow-up, outcome relevant baseline risk factors) for each pairwise comparison. We will present the available direct comparisons between different dietary interventions and control group using a network diagram for each outcome [38]. The size of the nodes will be proportional to the sample size to each dietary intervention and the thickness of the lines proportional to number of studies available. We will also use the contribution matrix to identify the direct comparisons with greater influence in the network relative effects [38, 39].

#### Standard pairwise meta-analyses and network meta-analyses

For each outcome measure of interest, random pairwise effects, and network meta-analyses will be performed in order to determine the pooled relative effect of each intervention relative to every other intervention in terms of the post-intervention values or the changes from baseline scores of the different dietary interventions. Separate pairwise meta-analyses will be used first to compare all the interventions with available direct evidence from at least two studies. Heterogeneity between trial results will be measured using the *I* ^2^ statistic, and *I* ^2^ > 50% will be considered to represent substantial heterogeneity. Forest plots will be generated to illustrate the study-specific effect sizes along with a 95% confidence interval (CI). Network meta-analysis will be then used to synthesize all the available evidence. Network meta-analysis methods are extensions of the standard pairwise meta-analysis model that enable a simultaneous comparison of multiple interventions forming a connected network while preserving the internal randomization of individual trials. We will perform a random effects network meta-analysis for each outcome to estimate all possible pairwise relative effects and we will obtain a clinically meaningful relative ranking of the different dietary interventions. We will present summary mean differences in a league table. We will also estimate the relative ranking of the different diets for each outcome using the distribution of the ranking probabilities, and the surface under the cumulative ranking curves (SUCRA) [40]. For each outcome, we will assume a common network-specific heterogeneity parameter, and we will estimate the predictive intervals to assess how much this heterogeneity affects the relative effects with respect to the additional uncertainty anticipated in future studies [41].

#### Assumption of transitivity

Transitivity is the fundamental assumption of indirect comparisons and network meta-analysis, and its violation threatens the validity of the findings obtained from a network of studies. We are considering the following effect modifiers: changes in body weight and mean baseline age. Exercise has been already defined as an exclusion criteria if not applied in intervention diets and control groups.

#### Assessment of inconsistency

To evaluate the presence of statistical inconsistency (i.e., disagreement between the different sources of evidence) in the data, we will employ both local and global approaches [42]. Specifically, we will use the loop-specific approach [43] to detect loops of evidence that might present important inconsistency as well as the node-splitting approach [44] to detect comparisons for which direct estimates disagree with indirect evidence from the entire network. Global methods investigate the presence of inconsistency jointly from all possible sources in the network. For this purpose we will use the design-by-treatment interaction model and the *I* ^2^ statistic [45, 46].

#### Subgroup and sensitivity analyses

In case of possible important heterogeneity or inconsistency, we will explore the possible sources using subgroup and meta-regression analyses. Subgroup analyses are planned for study length, sample size, age, sex, presence of comorbidities, and type of pharmaceutical intervention. We will assess the sensitivity of results for the primary outcome by analyzing only studies considered being at low risk of bias.

#### Small study effects and publication bias

When a sufficient number of studies are available (10 or more studies), we will use the comparison-adjusted funnel plot [38] to assess the presence of small-study effects in the network and contour-enhanced funnel plots [47], to investigate whether funnel plot asymmetry is likely to be explained by publication bias. We draw inference on the risk for publication bias based primarily on non-statistical considerations; hence, by considering how likely it is that studies may have been conducted but not published based on the expertise of the investigators in the field.

We will fit all analyses described in a frequentist framework using Stata 14.0 [48] (*network* package [49]), and we will produce presentation tools with the *network graphs* package [50].

## Quality of the evidence

We will first use our recently developed NutriGrade tool to evaluate and judge the meta-evidence for pairwise comparisons, which has been especially developed for nutrition research to address specific requirements for this research field [51]. Then, to infer about the quality of evidence from the network meta-analysis, we will combine our judgment about the direct comparisons with their contributions in the estimation within the network, as described in Salanti et al. [42].

## Discussion

This systematic review and network meta-analysis will be the first to summarize and to compare the effects of different dietary approaches on glycemic control and cardiovascular risk factors, using both direct and indirect evidence. This analysis will show which dietary interventions, if any, might be the most promising in the management of T2DM. The validity of the results will be limited by the heterogeneity of the different dietary interventions. A priori dietary patterns such as the Mediterranean diet or DASH are most likely assessed via different scoring systems. Likewise, diets defined by specific macronutrients will vary with respect to the exact percentage of fat, carbohydrates, or proteins in total energy consumption as well as the composition of the remaining macronutrients, respectively. Nevertheless, we are confident that this network meta-analysis will contribute to the methodological progress in the field of systematic reviews, since it will be the first to compare the direct and indirect effects of different dietary approaches in the management of T2DM. The results of the present network meta-analysis will influence evidence-based treatment decisions in, since it will be fundamental for based recommendations in the management of T2DM.

## Additional file

Additional file 1: PRISMA-P checklist. (DOCX 36 kb)

## Abbreviations

CI: Confidence interval
HbA1c: Glycosylated hemoglobin
T2DM: Type 2 diabetes mellitus

## References

[1] WHO. Global status report on noncommunicable diseases 2014. Geneva: World Health Organization; 2012.

[2] WHO (2013). Fact Sheet No. 312: Diabetes.

[3] American Diabetes Association (2016). 3. Foundations of Care and Comprehensive Medical Evaluation. Diabetes Care.

[4] Evert AB, Boucher JL, Cypress M, Dunbar SA, Franz MJ, Mayer-Davis EJ, Neumiller JJ, Nwankwo R, Verdi CL, Urbanski P (2014). Nutrition therapy recommendations for the management of adults with diabetes. Diabetes Care.

[5] Schwingshackl L, Hoffmann G (2014). Comparison of the long-term effects of high-fat v. low-fat diet consumption on cardiometabolic risk factors in subjects with abnormal glucose metabolism: a systematic review and meta-analysis. Br J Nutr.

[6] Schwingshackl L, Strasser B, Hoffmann G (2011). Effects of monounsaturated fatty acids on glycaemic control in patients with abnormal glucose metabolism: a systematic review and meta-analysis. Ann Nutr Metab.

[7] Schwingshackl L, Strasser B (2012). High-MUFA diets reduce fasting glucose in patients with type 2 diabetes. Ann Nutr Metab.

[8] Schwingshackl L, Hoffmann G. Monounsaturated fatty acids and risk of cardiovascular disease: synopsis of the evidence available from systematic reviews and meta-analyses. Nutrients. 2012;4(12):1989–2007. 10.3390/nu4121989 PMC3546618 23363996

[9] American Diabetes Association (2017). 4. Lifestyle Management. Diabetes Care.

[10] American Diabetes Association (2017). 9. Cardiovascular Disease and Risk Management. Diabetes Care.

[11] Ajala O, English P, Pinkney J (2013). Systematic review and meta-analysis of different dietary approaches to the management of type 2 diabetes. Am J Clin Nutr.

[12] Brand-Miller JC, Petocz P, Colagiuri S (2003). Meta-analysis of low-glycemic index diets in the management of diabetes: response to Franz. Diabetes Care.

[13] Kirk JK, Graves DE, Craven TE, Lipkin EW, Austin M, Margolis KL (2008). Restricted-carbohydrate diets in patients with type 2 diabetes: a meta-analysis. J Am Diet Assoc.

[14] Moher D, Shamseer L, Clarke M, Ghersi D, Liberati A, Petticrew M, Shekelle P, Stewart LA, Group P-P (2015). Preferred reporting items for systematic review and meta-analysis protocols (PRISMA-P) 2015 statement. Syst Rev.

[15] Shamseer L, Moher D, Clarke M, Ghersi D, Liberati AD, Petticrew M, Shekelle P, Stewart LA, the P-PG (2015). Preferred reporting items for systematic review and meta-analysis protocols (PRISMA-P) 2015: elaboration and explanation. BMJ.

[16] Hutton B, Salanti G, Caldwell DM, Chaimani A, Schmid CH, Cameron C, Ioannidis JP, Straus S, Thorlund K, Jansen JP (2015). The PRISMA extension statement for reporting of systematic reviews incorporating network meta-analyses of health care interventions: checklist and explanations. Ann Intern Med.

[17] Chaimani A, Caldwell DM, Li T, Higgins JP, Salanti G: Additional considerations are required when preparing a protocol for a systematic review with multipleinterventions. J Clin Epidemiol 2017. (epub ahead of print). 10.1016/j.jclinepi.2016.11.015 28088593

[18] American Diabetes Association (2015). (2) Classification and diagnosis of diabetes. Diabetes Care.

[19] American Diabetes Association (2017). 2. Classification and Diagnosis of Diabetes. Diabetes Care.

[20] Schwingshackl L, Hoffmann G (2013). Comparison of effects of long-term low-fat vs high-fat diets on blood lipid levels in overweight or obese patients: a systematic review and meta-analysis. J Acad Nutr Diet.

[21] Schwingshackl L, Hoffmann G (2013). Long-term effects of low-fat diets either low or high in protein on cardiovascular and metabolic risk factors: a systematic review and meta-analysis. Nutr J.

[22] Schwingshackl L, Hoffmann G (2014). Mediterranean dietary pattern, inflammation and endothelial function: a systematic review and meta-analysis of intervention trials. Nutr Metab Cardiovasc Dis.

[23] Schwingshackl L, Hoffmann G (2014). Adherence to Mediterranean diet and risk of cancer: a systematic review and meta-analysis of observational studies. Int J Cancer.

[24] Schwingshackl L, Missbach B, Konig J, Hoffmann G (2015). Adherence to a Mediterranean diet and risk of diabetes: a systematic review and meta-analysis. Public Health Nutr.

[25] Schwingshackl L, Hoffmann G (2016). Does a Mediterranean-Type Diet Reduce Cancer Risk?. Curr Nutr Rep.

[26] Schwingshackl L, Hoffmann G (2015). Adherence to Mediterranean diet and risk of cancer: an updated systematic review and meta-analysis of observational studies. Cancer Med.

[27] Appel LJ, Brands MW, Daniels SR, Karanja N, Elmer PJ, Sacks FM (2006). Dietary approaches to prevent and treat hypertension: a scientific statement from the American Heart Association. Hypertension.

[28] Schwingshackl L, Hoffmann G (2013). Long-term effects of low glycemic index/load vs. high glycemic index/load diets on parameters of obesity and obesity-associated risks: a systematic review and meta-analysis. Nutr Metab Cardiovasc Dis.

[29] Schwingshackl L, Hobl LP, Hoffmann G (2015). Effects of low glycaemic index/low glycaemic load vs. high glycaemic index/high glycaemic load diets on overweight/obesity and associated risk factors in children and adolescents: a systematic review and meta-analysis. Nutr J.

[30] Schwingshackl L, Missbach B, Dias S, Konig J, Hoffmann G (2014). Impact of different training modalities on glycaemic control and blood lipids in patients with type 2 diabetes: a systematic review and network meta-analysis. Diabetologia.

[31] Schwingshackl L, Dias S, Strasser B, Hoffmann G (2013). Impact of different training modalities on anthropometric and metabolic characteristics in overweight/obese subjects: a systematic review and network meta-analysis. PLoS One.

[32] Wharton S, Sharma AM, Lau DC (2013). Weight management in diabetes. Can J Diabetes.

[33] American Diabetes Association (2017). 7. Obesity Management for the Treatment of Type 2 Diabetes. Diabetes Care.

[34] Lyons TJ, Basu A (2012). Biomarkers In Diabetes: Hemoglobin A1c, Vascular and tissue markers. Transl Res.

[35] van Holten TC, Waanders LF, de Groot PG, Vissers J, Hoefer IE, Pasterkamp G, Prins MW, Roest M (2013). Circulating biomarkers for predicting cardiovascular disease risk: a systematic review and comprehensive overview of meta-analyses. PLoS One.

[36] Higgins JP, Altman DG, Gotzsche PC, Juni P, Moher D, Oxman AD, Savovic J, Schulz KF, Weeks L, Sterne JA (2011). The Cochrane Collaboration’s tool for assessing risk of bias in randomised trials. BMJ.

[37] Higgins JPT, Green S (editors). Cochrane Handbook for Systematic Reviews of Interventions Version 5.1.0 [updated March 2011]. The Cochrane Collaboration, 2011. Available from www.cochrane-handbook.org. Accessed 20 Aug 2016.

[38] Chaimani A, Higgins JP, Mavridis D, Spyridonos P, Salanti G (2013). Graphical tools for network meta-analysis in STATA. PLoS One.

[39] Krahn U, Binder H, Konig J (2013). A graphical tool for locating inconsistency in network meta-analyses. BMC Med Res Methodol.

[40] Salanti G, Ades AE, Ioannidis JP (2011). Graphical methods and numerical summaries for presenting results from multiple-treatment meta-analysis: an overview and tutorial. J Clin Epidemiol.

[41] Riley RD, Higgins JP, Deeks JJ (2011). Interpretation of random effects meta-analyses. BMJ.

[42] Salanti G, Del Giovane C, Chaimani A, Caldwell DM, Higgins JP (2014). Evaluating the quality of evidence from a network meta-analysis. PLoS One.

[43] Bucher HC, Guyatt GH, Griffith LE, Walter SD (1997). The results of direct and indirect treatment comparisons in meta-analysis of randomized controlled trials. J Clin Epidemiol.

[44] Dias S, Welton NJ, Caldwell DM, Ades AE (2010). Checking consistency in mixed treatment comparison meta-analysis. Stat Med.

[45] Higgins JP, Jackson D, Barrett JK, Lu G, Ades AE, White IR (2012). Consistency and inconsistency in network meta-analysis: concepts and models for multi-arm studies. Res Synth Methods.

[46] Jackson D, Barrett JK, Rice S, White IR, Higgins JP (2014). A design-by-treatment interaction model for network meta-analysis with random inconsistency effects. Stat Med.

[47] Peters JL, Sutton AJ, Jones DR, Abrams KR, Rushton L (2008). Contour-enhanced meta-analysis funnel plots help distinguish publication bias from other causes of asymmetry. J Clin Epidemiol.

[48] StataCorp (2015). Stata Statistical Software: Release 14.

[49] White IR. Network meta-analysis. Stata J. 2015;15(4):951-85.

[50] Chaimani, Salanti. Visualizing assumptions and results in network meta-analysis: the network graphs package. Stata J. 2015;15(4):905-50.

[51] Schwingshackl L, Knüppel S, Schwedhelm C, Hoffmann G, Missbach B, Stelmach-Mardas M, Dietrich S, Eichelmann F, Kontopanteils E, Iqbal K (2016). Perspective: NutriGrade: a scoring system to assess and judge the meta-evidence of randomized controlled trials and cohort studies in nutrition research. Adv Nutr.

